# Reproducibility and validity of the food frequency questionnaire for estimating habitual dietary intake in children and adolescents

**DOI:** 10.1186/1475-2891-10-27

**Published:** 2011-03-24

**Authors:** Tomomi Kobayashi, Miharu Kamimura, Shino Imai, Chihiro Toji, Naoko Okamoto, Mitsuru Fukui, Chigusa Date

**Affiliations:** 1 Department of Food and Nutritional Sciences, School of Natural Science and Ecological Awareness, Graduate School of Humanities and Sciences, Nara Women’s University, Kitauoya-nishimachi, Nara, 630-8506, Japan; 2 Department of Food Science and Nutrition, Faculty of Human Environmental Sciences, Mukogawa Women's University, 6-46 Ikebiraki-cho, Nishinomiya, Hyogo 663-8558, Japan; 3 Sanpoku Primary School in Murakami City, 526 Horinouchi, Murakami, Niigata, 959-3905, Japan; 4 Department of Food Sciences and Nutrition, Faculty of Human Life and Environment, Nara Women’s University, Kitauoya-nishimachi, Nara, 630-8506, Japan; 5 Department of Food Science and Nutrition, Faculty of Wellness, Shigakukan university, 55 Nadakayama, Yokone-cho, Daifu, Aichi, 474-8651, Japan; 6 Laboratory of Statistics, School of Medicine, Osaka City University, 1-4-3 Asahi-machi, Abeno-ku, Osaka, 545-8585, Japan; 7 School of Human Science and Environment, University of Hyogo, 1-1-12 Motomachi-cho, Shinzaike, Himezi, 670-0092, Japan

## Abstract

**Background:**

A previous study reported the development a 75-item food frequency questionnaire for Japanese children (CFFQ). The first aim was to examine the reproducibility and validity of the CFFQ in order to assess dietary intake among two groups; 3-11 year old children (YC group) and 12-16 year old children (AD group). The second aim was to use the CFFQ and the FFQ for adults (AFFQ), and to determine which was better suited for assessing the intake of children in each group.

**Methods:**

A total of the 103 children participated in this study. The interval between the first CFFQ and AFFQ and the second CFFQ and AFFQ was one month. Four weighted dietary records (WDRs) were conducted once a week. Pearson's correlation coefficients between the first and second FFQs were calculated to test the reproducibility of each FFQ. Pearson's correlation coefficients between WDRs and the second FFQ were calculated for the unadjusted value and sex-, age-, and energy-adjusted values to determine the validity of each FFQ.

**Results:**

The final number of subjects participating in the analysis was 89. The median correlation coefficients between the first and second CFFQs and AFFQs were 0.76 and 0.73, respectively. There was some over/underestimation of nutrients in the CFFQ of the YC group and in the AFFQ of the AD group. The medians of the sex-, age-, and energy-adjusted correlation coefficients were not different between the YC and AD groups for each FFQ. The correlation coefficient in sex-, age-, and energy-adjusted value revealed that the largest number of subject with high (0.50 or more) value was obtained by the CFFQ in the YC group.

**Conclusions:**

This study indicated that the CFFQ might be a useful tool for assessing habitual dietary intake of children in the YC group. Although the CFFQ agreed moderately with habitual intake, it was found to underestimate intake in theAD group. However, for the AFFQ, the ability to rank habitual intake was low. Therefore, it is necessary to develop a new FFQ or modify an existing FFQ to accurately assess the habitual diet of children in the AD group.

## Background

It is thought that childhood is not only the major period of growth, but is also the time when eating habits are formed [1]. An inactive lifestyle and long-term eating habits such as irregularity and overeating affect the initiation and onset of lifestyle-related diseases, such as obesity, diabetes, and cardiovascular disease. In order to prevent these diseases, it is desirable that individuals should acquire appropriate dietary habits during childhood. We cannot conduct weighed dietary records (WDRs) for extended periods of time for children because keeping a WDR puts a heavy burden on the participant's parents as surrogates. It is also not feasible to conduct 24-hour recall for multiple days in order to assess children's habitual dietary intake, because it is difficult to obtain accurate dietary information because children lack knowledge about foods and cooking methods [2-4]. Although the quantitative assessment of food intake using a food frequency questionnaire (FFQ) may be less accurate compared with a WDR and 24-hour recall, it is a method that can assess the habitual dietary patterns of many subjects, even from a one-time investigation.

In many epidemiological studies, a FFQ originally developed for adults was applied for the assessment of dietary intake in children [5-8]. We thought that a dietary survey method for children was more suitable to assess their dietary intake than the method developed for adults. However, there have been no reports of the validity of the conventional FFQ for adults or of the FFQ for children in the Japanese pediatric population [9]. To prove our hypothesis, we previously developed a 75-item FFQ (CFFQ) to assess the habitual diets of Japanese children [10]. This CFFQ included the average portion size consumed by 3 to 11-year-old children, including both the individual foods and the items in mixed dishes. However, the reproducibility and validity of this questionnaire in the target group needed to be examined. In addition, we wanted to confirm the validity of the FFQ in adolescents, because there have been no FFQs developed for adolescents in Japan [9].

The aims of this study were two-fold. The first aim was to examine the reproducibility and validity of the CFFQ in order to assess dietary intakes among two groups of children divided by age:, 3 to 11 year old children (YC group) and 12 to 16 year old children (AD group). The second aim was to determine more suitable FFQ for assessing the children's intake in both groups. To accomplish this, we applied the CFFQ and the FFQ for adults (AFFQ), which was previously developed for dietary assessment in adults by Date et al. [11], for the two groups of children.

## Methods

### Subjects

The study participants were healthy children enrolled at the kindergarten, elementary school, and secondary school attached to Nara Women's University. A total of 111 participants were chosen by the teachers of each school. We grouped the participants into a "Young Children" group (YC group; 3-11 years) and an "Adolescent" group (AD group; 12-16 years). We explained the purpose of the investigation and the methods to the parents/guardians of the YC group in May and June 2008, and to the participants in the AD group in October of the same year. A total of 103 parents/guardians (YC group n = 50, AD group n = 53) gave written informed consent for their children to participate in this study. The study was approved by the research ethics committee of the faculty of human life and environment, Nara Women's University.

### Study design

**Figure 1:**
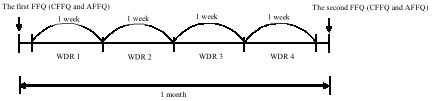
**Design of the food frequency questionnaire reproducibility and validity study**. The order of the CFFQ and AFFQ was random for each subject. We applied the FFQs and 4-day WDRs to 50 subjects in the YC group and 53 in the AD group. There were 89 subjects who completed all FFQs and the 4-day WDRs (YC group n = 48, AD group n = 41). CFFQ: Food frequency questionnaire for dietary assessment in children. AFFQ: Food frequency questionnaire for dietary assessment in adults. WDR: Weighed dietary record.

Figure 1 shows the design of the study. The two types of FFQs (the CFFQ and AFFQ) were conducted at the same time in random order for each subject. The first FFQs were conducted just before performing the first WDR. Then, the four-day WDRs were collected once a week, and on each different day of the week (3 weekdays and 1 weekend day). We initially allocated the days of the dietary survey for each subject at random. The second FFQs were conducted after the fourth WDR. The interval between the first FFQs and the second FFQs was one month.

### Food frequency questionnaires

The FFQs were completed by the subjects' mothers in the YC group. In the AD group, the subjects self-administered the FFQs. We used two FFQs in this study. The two FFQs included questions about both individual food items and mixed dishes based upon the typical eating habits of normal Japanese children or adults.

The first was the CFFQ which was developed specifically for Japanese children. Details of the CFFQ were reported previously [10]. In brief, the CFFQ is a newly developed questionnaire for assessing the habitual dietary intake of children during the previous month. The question items and portion size of the CFFQ were developed from the dietary data of 586 children (age: 3 to 11-years-old). The number of food items on the CFFQ is 75.

The second was the AFFQ, which was developed for estimating the habitual dietary intake of Japanese adults [11]. The AFFQ is composed the 76-food items. The portion size of the AFFQ is tailored to the average of the dietary intakes of adults. We did not change the portion size in this study. Two items concerning alcohol intake were excluded, leaving 74 items on the AFFQ. The AFFQ was developed to report the adults' recall of their diets over the past year. In this study, the frequency response formats were modified to encompass the past month for each child's diet. In Japan, children are provided with lunch in elementary school on weekdays. Therefore, questions about school lunch were added in the AFFQ.

For both the CFFQ and AFFQ, the intake frequencies of the each question item on the FFQs were classified into four types: seven (i.e., everyday, 5-6 times per week, 3-4 times per week, 1-2 times per week, 2-3 times per month, 1 time per month, or never), eight ("2-3 times per day" was added to seven categories), nine ("4-5 times per day" was added to eight categories) and eleven ("8-10 times per day", "6-7 times per day" were added to nine categories) according to the general intake frequency of each item. We used photographs of each listed food item to estimate one portion. The estimation of portion size was classified into six categories referring to the photographs in full-scale size; that is, one-third, one-half, the same amount, 1.5 times, twice, and 'other'.

### Weighed dietary records

We decided that the subjects' mothers would keep the WDRs for the YC group, and either the subjects or their mothers would keep them in the AD group. In order to measure the weight of foods, a digital cooking scale (TANITA digital cooking scale; KD-402-WH, Tokyo, Japan) was loaned to the subjects for the duration of the study. On a specified day, the weight of all foods and beverages that the subjects consumed, from getting up until going to bed, was measured. The dish name, food name and weight were recorded for each meal (breakfast, lunch, dinner and snacks). We asked the respondents to measure the weight of foods before cooking, as much as they could. The mean value of the four WDRs was defined as the subject's habitual intake for one month.

### Calculation of nutrient intake

After the data from the first and second FFQs and the 4-day WDRs were collected, registered dieticians confirmed the content of the written survey forms. Any unclear points were inquired of the respondents, and then FFQs and WDRs were completed. We then calculated the nutrient intake for each child using the Standard Tables of Food Composition in Japan (fifth revised and enlarged edition) [12]. We calculated energy intake and intake of 26 nutrients (protein, total fat, carbohydrate, sodium, potassium, calcium, magnesium, phosphorous, iron, zinc, copper, manganese, retinol equivalent, vitamin D, alpha tocopherol, vitamin K, vitamins B1, vitamins B2, niacin, vitamin B6, vitamin B12, folic acid, pantothenic acid, vitamins C, cholesterol, dietary fiber), and 12 fatty acids (saturated fatty acid, monounsaturated fatty acid, polyunsaturated fatty acid, n-3 polyunsaturated fatty acid, n-6 polyunsaturated fatty acid, myristic acid, oleic acid, linoleic acid, alpha - linolenic acid, arachidonic acid, icosapentaenoic acid, docosahexaenoic acid) from the WDRs and FFQs.

### Statistical analysis

The sample size was calculated to be appropriate for each group (p < 0.05, 80% power). We considered that the mean value of nutrients from the four WDRs was the gold standard (follow WDRs). In order to test the reproducibility of the FFQs, the Pearson's correlation coefficient between the first and second FFQs was calculated. To test the validity of the FFQs, we calculated the percent difference of intake between the WDRs and the second FFQ, using the following formula: (the second FFQ - WDRs)/WDRs. The paired t-test was used to examine the difference in nutrient intake between the WDRs and the second FFQ. Pearson's correlation coefficients between the WDRs and the second FFQ were calculated for the unadjusted (crude value) and sex-, age-, and energy-adjusted values (adjusted value). Energy was adjusted by the residual method [13]. A Bland Altman analysis was used to assess the agreement of the measurements between the CFFQ and WDR or the AFFQ and WDR [14]. The differences between the two methods were plotted against the mean of the two methods. Desirable agreement between two methods would result in a difference of 0.

We found that some nutrients were not normally distributed, so we calculated the natural log of these variables, attempting to correct for their non-normally. The statistical package SPSS for Windows 17.0 (SPSS Inc. Tokyo, Japan) was used for all statistical analyses.

## Results

### Subjects

Out of the 103 study participants, we excluded two individuals who did not eat their daily diet on the survey days; three who only kept WDRs for less than 2 days; and six who could not complete four FFQs (i.e, both the first and the second CFFQ and the AFFQ). Furthermore, using the cut-off value proposed by Willett [8], we excluded three subjects whose energy intake from the second CFFQ or AFFQ was <800 kcal or >4000 kcal for boys, and <500 kcal or >3500 kcal for girls. Therefore, the final number of subjects included for analysis was 89 (YC group n = 48, AD group n = 41). There were 15 children in kindergarten (3-5 years old), 18 in the lower grades of elementary school (6-8 years old), 15 in the upper grades of elementary school (9-11 years old), 24 junior high students (12-14 years old), and 17 high school students (15-16 years old).

### Reproducibility of the CFFQ and AFFQ

Table 1 shows the Pearson's correlation coefficients for total energy, nutrients and fatty acids between the first and second CFFQs, and the first and second AFFQs for all of the subjects. The correlation coefficients varied from 0.67 for carbohydrate to 0.84 for pantothenic acid in the CFFQ, and from 0.39 for manganese to 0.83 for carbohydrate in the AFFQ. The median correlation coefficient was 0.76 in the CFFQ and 0.73 in the AFFQ. For both the CFFQ and AFFQ, there were significant positive associations for the energy and 26 nutrients and 12 fatty acids between the first and second FFQs.

**Table 1:** Pearson's correlation coefficient between the first and second FFQs for all subjects

Nutrients	CFFQ	AFFQ
	n = 89	n = 89
Energy (kcal/day)	0.73**	0.80**
Protein (g/day)	0.77**	0.73**
Total fat (g/day)	0.76**	0.71**
Carbohydrate (g/day)	0.67**	0.83**
Sodium (mg/day)	0.76**	0.75**
Potassium (mg/day)	0.77**	0.72**
Calcium (mg/day)	0.78**	0.78**
Magnesium (mg/day)	0.77**	0.79**
Phosphorous (mg/day)	0.79**	0.77**
Iron (mg/day)	0.74**	0.67**
Zinc (mg/day)	0.79**	0.76**
Copper (mg/day)	0.69**	0.75**
Manganese (mg/day)	0.72**	0.39**
Vitamin A (μgRE/day)	0.72**	0.72**
Vitamin D (mcg/day)	0.74**	0.65**
Alpha tocopherol (mg/day)^‡^	0.76**	0.73**
Vitamin K (mg/day)^‡^	0.82**	0.68**
Vitamins B1 (mg/day)^‡^	0.80**	0.78**
Vitamins B2 (mg/day)^‡^	0.82**	0.77**
Niacin (mg/day)^‡^	0.80**	0.77**
Vitamin B6 (mg/day)^‡^	0.82**	0.77**
Vitamin B12 (μg/day)^‡^	0.76**	0.71**
Folic acid (μg/day)^‡^	0.81**	0.67**
Pantothenic acid (mg/day)^‡^	0.84**	0.79**
Vitamins C (mg/day)^‡^	0.72**	0.61**
Cholesterol (mg/day)^‡^	0.80**	0.67**
Dietary fiber (g/day)^‡^	0.78**	0.77**
Saturated fatty acid (g/day)^‡^	0.81**	0.80**
Monounsaturated fatty acid (g/day)^‡^	0.78**	0.74**
Polyunsaturated fatty acid (g/day)^‡^	0.78**	0.72**
n-3 polyunsaturated fatty acid (mg/day)^‡^	0.56**	0.72**
n-6 polyunsaturated fatty acid (mg/day)^‡^	0.55**	0.71**
Myristic acid (mg/day)^‡^	0.71**	0.82**
Oleic acid (mg/day)^‡^	0.57**	0.74**
Linoleic acid (mg/day)^‡^	0.54**	0.71**
Alpha-linolenic acid (mg/day)^‡^	0.58**	0.70**
Arachidonic acid (mg/day)^‡^	0.61**	0.68**
Icosapentaenoic acid (mg/day)^‡^	0.56**	0.63**
Docosahexaenoic acid (mg/day)^‡^	0.55**	0.65**

‡: The data (Alpha tocopherol, Vitamin K, Vitamins B1, B2, Niacin, B6, and B12) were transformed by natural log to improve normality

CFFQ: Food frequency questionnaire for dietary assessment in children

AFFQ: Food frequency questionnaire for dietary assessment in adults

**: p < 0.01

### Validity of the second CFFQ

Table 2 shows the intakes of total energy, nutrients and fatty acids determined from the WDRs, and from the second CFFQ in the YC and AD groups. The mean energy intake was not significantly different between the WDRs (1,547 ± 330 kcal/day; i.e. mean ± SD) and the second CFFQ (1,533 ± 438 kcal/day) in the YC group. However the energy intake was found to be underestimated for the second CFFQ (1,781 ± 588 kcal/day) compared to the WDRs (2,078 ± 478 kcal/day) in the AD group. The median percent difference between the WDRs and CFFQ of nutrients were -2% in the YC group and -18% in the AD group, respectively. Table 3 shows the correlation coefficients of energy, nutrients and fatty acids between WDRs and the second CFFQ in the YC and AD groups. From the CFFQ, the median of the crude values for nutrients was 0.55 in the YC group and 0.28 in the AD group. The adjusted values varied from 0.03 for vitamin C to 0.69 for magnesium in the YC group (the median; r = 0.39), and from 0.15 for monounsaturated fatty acid and oleic acid to 0.77 for vitamin C in the AD group (the median; r = 0.34).

**Table 2:** Intake of nutrients assessed by WDR, CFFQ and AFFQ for young children and adolescents

Nutrients	Young children (n = 48)								Adolescents (n = 41)							
		
	WDR		CFFQ			AFFQ			WDR		CFFQ			AFFQ		
						
	Mean	SD	Mean	SD	% difference^†^	Mean	SD	% difference^†^	Mean	SD	Mean	SD	% difference^†^	Mean	SD	% difference^†^
Energy (kcal/day)	1547	330	1533	438	-1	1757	538	14**	2078	478	1781	588	-14**	2257	643	9
Protein (g/day)	58.2	14.2	57.9	20.4	0	68.5	24.3	18**	75.6	16.9	61.8	24.0	-18**	80.6	24.3	7
Total fat (g/day)	52.8	13.7	51.7	19.1	-2	63.0	24.8	20**	70.2	16.2	54.8	21.9	-22**	74.5	24.3	6
Carbohydrates (g/day)	205.5	49.7	204.8	51.6	0	223.3	57.2	9*	277.6	76.4	255.5	81.9	-8^ns^	308.5	91.4	11*
Sodium (mg/day)	3004	933	2509	865	-16**	3381	1316	13*	3893	1132	2509	930	-36**	4140	1477	6
Potassium (mg/day)	1924	462	1924	637	0	2365	862	23**	2348	616	2129	1053	-9^ns^	2897	1114	23**
Calcium (mg/day)	520	160	546	238	5	615	289	18*	540	172	605	479	12^ns^	702	363	30**
Magnesium (mg/day)	201	56	192	61	-5	236	80	17**	238	64	223	98	-6^ns^	293	102	23**
Phosphorous (mg/day)	920	225	928	323	1	1074	392	17**	1093	238	1021	488	-7^ns^	1269	421	16*
Iron (mg/day)	6.3	2.8	5.4	1.8	-13*	7.3	2.5	16*	7.9	2.7	6.1	2.3	-23**	8.8	2.9	11
Zinc (mg/day)	7.1	1.9	7.6	2.5	7	8.3	2.9	17**	9.6	2.5	8.2	3.0	-14**	10.3	3.2	7
Copper (mg/day)	0.93	0.29	0.87	0.25	-6	1.07	0.31	15**	1.16	0.30	1.05	0.36	-10^ns^	1.35	0.40	16**
Manganese (mg/day)	2.27	0.94	2.34	0.68	3	3.61	1.50	59**	3.02	1.08	2.81	0.89	-7^ns^	4.24	1.59	40**
Vitamin A (μgRE/day)	490	230	466	174	-5	560	226	14*	602	350	516	280	-14^ns^	675	274	12
Vitamin D (μg/day)	6.0	4.5	4.9	2.3	-18**	6.2	2.5	3	7.5	4.5	5.8	3.8	-22^ns^	7.1	2.6	-5
Alpha tocopherol (mg/day)^‡^	5.7	1.7	4.9	1.7	-15**	6.9	2.5	20**	7.5	2.4	5.2	1.9	-31**	8.4	2.7	11
Vitamin K (μg/day)^‡^	179	134	142	61	-21	190	83	6	223	94	130	71	-42**	221	106	-1
Vitamins B1 (mg/day)^‡^	0.79	0.21	0.77	0.26	-2	0.89	0.32	13*	1.00	0.31	0.84	0.35	-16*	1.07	0.33	7
Vitamins B2 (mg/day)^‡^	1.04	0.29	1.08	0.41	3	1.40	0.54	35**	1.26	0.33	1.20	0.72	-5^ns^	1.60	0.66	27**
Niacin (mg/day)^‡^	11.3	3.5	11.2	4.4	0	14.3	5.4	27**	14.8	4.0	11.9	4.2	-20**	17.0	5.5	15*
Vitamin B6 (mg/day)^‡^	0.91	0.26	0.90	0.33	-1	1.15	0.43	26**	1.20	0.35	1.00	0.40	-17**	1.42	0.49	18*
Vitamin B12 (μg/day)^‡^	4.3	3.9	4.8	1.9	12	6.3	2.5	46**	6.5	4.3	5.5	2.5	-15^ns^	7.3	2.6	12
Folic acid (μg/day)^‡^	228	73	215	68	-6	330	122	45**	284	95	220	81	-23**	386	142	36**
Pantothenic acid (mg/day)^‡^	5.15	1.38	5.19	1.80	1	6.05	2.17	17**	6.43	1.44	5.89	2.78	-8^ns^	7.38	2.46	15*
Vitamins C (mg/day)^‡^	64	23	60	22	-6	99	38	55**	90	61	78	40	-14^ns^	132	53	47**
Cholesterol (mg/day)^‡^	311	112	280	116	-10	323	126	4	421	119	300	122	-29**	403	147	-4
Dietary fiber (g/day)^‡^	11.1	3.3	9.5	3.0	-15**	11.8	4.2	7	13.6	4.5	10.3	3.9	-24**	14.8	4.8	9
Saturated fatty acid (g/day)^‡^	17.00	5.00	18.36	7.20	8	21.25	8.97	25**	22.00	6.07	19.53	9.41	-11^ns^	24.56	9.58	12
Monounsaturated fatty acid (g/day)^‡^	19.05	5.28	18.05	6.93	-5	22.14	9.01	16*	26.20	6.39	19.25	7.28	-27**	26.45	8.52	1
Polyunsaturated fatty acid (g/day)^‡^	10.41	3.44	9.22	3.23	-11*	12.35	4.68	19*	13.66	3.63	9.58	3.45	-30**	14.82	4.64	8
n-3 polyunsaturated fatty acid (mg/day)^‡^	1.50	0.58	1.43	0.56	-4	1.98	0.76	32**	2.21	0.77	1.55	0.57	-30**	2.39	0.80	8
n-6 polyunsaturated fatty acid (mg/day)^‡^	8.90	3.02	7.78	2.72	-13*	10.34	3.94	16*	11.42	3.09	8.01	2.91	-30**	12.40	3.87	9
Myristic acid (mg/day)^‡^	1339	614	1616	780	21**	1882	983	41**	1649	667	1659	1120	1^ns^	1986	1058	20*
Oleic acid (mg/day)^‡^	17609	4901	16503	6302	-6	20310	8257	15*	23808	5865	17614	6704	-26**	24323	7838	2
Linoleic acid (mg/day)^‡^	8652	2986	7520	2619	-13*	10027	3814	16*	11080	3049	7741	2820	-30**	12027	3752	9
Alpha-linolenic acid (mg/day)^‡^	1082	397	959	342	-11*	1403	563	30**	1509	510	1008	400	-33**	1717	585	14
Arachidonic acid (mg/day)^‡^	136	46	136	56	0	158	62	16*	193	49	144	55	-25**	191	67	-1
Icosapentaenoic acid (mg/day)^‡^	112	125	138	90	23	167	76	49**	179	147	154	69	-14^ns^	189	81	5
Docosahexaenoic acid (mg/day)^‡^	227	195	243	151	7	301	135	33**	376	266	284	126	-25*	361	143	-4

†: (the second FFQ - WDR)/WDR (%)

‡: The data were transformed by natural log to improve normality

WDR: Weighed dietary record

CFFQ: Food frequency questionnaire for dietary assessment in children

AFFQ: Food frequency questionnaire for dietary assessment in adults

SD: Standard deviation

Paired t test *p < 0.05, **: p < 0.01

**Table 3:** Correlation coefficients of nutrients between the WDRs and the CFFQ for young children and adolescents

Nutrients	Young children (n = 48)		Adolescents (n = 41)	
		
	crude value^a^	adjusted value^b^	crude value^a^	adjusted value^b^
Energy (kcal/day)	0.66**		0.33*	
Protein (g/day)	0.66**	0.19	0.37*	0.23
Total fat (g/day)	0.51**	0.50**	0.14	0.19
Carbohydrate (g/day)	0.66**	0.38**	0.39*	0.22
Sodium (mg/day)	0.40**	0.17	0.23	0.26
Potassium (mg/day)	0.63**	0.45**	0.24	0.44**
Calcium (mg/day)	0.58**	0.34*	0.28	0.26
Magnesium (mg/day)	0.60**	0.69**	0.13	0.19
Phosphorous (mg/day)	0.65**	0.29*	0.30	0.15
Iron (mg/day)	0.47**	0.16	0.20	0.47**
Zinc (mg/day)	0.59**	0.21	0.33*	0.16
Copper (mg/day)	0.59**	0.25	0.24	0.42**
Manganese (mg/day)	0.73**	0.59**	0.31*	0.36*
Vitamin A (μgRE/day)	0.59**	0.27	0.31*	0.34*
Vitamin D (μg/day)	0.43**	0.33*	0.15	0.20
Alpha tocopherol (mg/day)^‡^	0.55**	0.40**	0.13	0.43**
Vitamin K (μg/day)^‡^	0.43**	0.41**	0.23	0.58**
Vitamins B1 (mg/day)^‡^	0.57**	0.31*	0.11	0.38*
Vitamins B2 (mg/day)^‡^	0.70**	0.48**	0.38*	0.40*
Niacin (mg/day)^‡^	0.61**	0.14	0.42**	0.46**
Vitamin B6 (mg/day)^‡^	0.70**	0.42**	0.40*	0.56**
Vitamin B12 (μg/day)^‡^	0.43**	0.16	0.40*	0.31
Folic acid (μg/day)^‡^	0.55**	0.47**	0.28	0.64**
Pantothenic acid (mg/day)^‡^	0.69**	0.43**	0.38*	0.37*
Vitamins C (mg/day)^‡^	0.33*	0.03	0.31*	0.77**
Cholesterol (mg/day)^‡^	0.47**	0.32*	0.34*	0.30
Dietary fiber (g/day)^‡^	0.56**	0.51**	0.22	0.47**
Saturated fatty acid (g/day)^‡^	0.60**	0.44**	0.16	0.47**
Monounsaturated fatty acid (g/day)^‡^	0.50**	0.41**	0.11	0.15
Polyunsaturated fatty acid (g/day)^‡^	0.46**	0.57**	0.07	0.26
n-3 polyunsaturated fatty acid (mg/day)^‡^	0.54**	0.56**	0.16	0.36*
n-6 polyunsaturated fatty acid (mg/day)^‡^	0.43**	0.56**	0.05	0.23
Myristic acid (mg/day)^‡^	0.68**	0.27	0.34*	0.44**
Oleic acid (mg/day)^‡^	0.48**	0.43**	0.06	0.15
Linoleic acid (mg/day)^‡^	0.43**	0.56**	0.04	0.23
Alpha-linolenic acid (mg/day)^‡^	0.37*	0.47**	-0.06	0.17
Arachidonic acid (mg/day)^‡^	0.50**	0.34*	0.42**	0.39*
Icosapentaenoic acid (mg/day)^‡^	0.48**	0.35*	0.30	0.34*
Docosahexaenoic acid (mg/day)^‡^	0.52**	0.37*	0.30	0.31

a: Unadjusted Pearson correlation coefficient

b: Nutrient intakes were adjusted for age, sex and energy

‡: The data (Alpha tocopherol, Vitamin K, Vitamin B1, Vitamin B2, Niacin, Vitamin B6, and Vitamin B12) were transformed by natural log to improve normality

CFFQ: Food frequency questionnaire for dietary assessment in children

AFFQ: Food frequency questionnaire for dietary assessment in adults

WDR: Weighed dietary record

SD: Standard deviation

*p < 0.05, **: p < 0.01

### Validity of the second AFFQ

There was the overestimate of the energy intake determined by the AFFQ in the YC group (1757 ± 538 kcal/day) (Table 2). However, there was no significant change in the energy intake calculated from the AFFQ in the AD group (2257 ± 643 kcal/day). The median percent difference between the WDRs and AFFQ with regard to nutrients was 17% in the YC group and 11% in the AD group. Table 4 shows the correlation coefficients of energy, nutrients and fatty acids between the WDRs and the second AFFQ in the YC and AD groups. In the AFFQ, the adjusted values varied from 0.01 for Alpha-linolenic acid to 0.68 for magnesium in the YC group (the median; r = 0.30), and from -0.01 for carbohydrate to 0.63 for Myristic acid in the AD group (the median; r = 0.29).

**Table 4:** Correlation coefficients of nutrients between the WDRs and the AFFQ for young children and adolescents

Nutrients	Young children (n = 48)		Adolescents (n = 41)	
		
	crude value^a^	adjusted value^b^	crude value^a^	adjusted value^b^
Energy (kcal/day)	0.57**		0.31	
Protein (g/day)	0.55**	0.15	0.25	0.32*
Total fat (g/day)	0.41**	0.23	-0.07	-0.01
Carbohydrate (g/day)	0.57**	0.17	0.45**	-0.01
Sodium (mg/day)	0.39**	0.41**	0.30	0.41**
Potassium (mg/day)	0.54**	0.53**	0.25	0.34*
Calcium (mg/day)	0.50**	0.33*	0.37*	0.50**
Magnesium (mg/day)	0.50**	0.68**	0.16	0.17
Phosphorous (mg/day)	0.54**	0.28	0.28	0.25
Iron (mg/day)	0.41**	0.27	0.25	0.44**
Zinc (mg/day)	0.49**	0.22	0.36*	0.05
Copper (mg/day)	0.51**	0.47**	0.40*	0.45**
Manganese (mg/day)	0.71**	0.61**	0.26	0.15
Vitamin A (μgRE/day)	0.25	0.42**	0.21	0.30
Vitamin D (μg/day)	0.23	0.24	0.17	0.26
Alpha tocopherol (mg/day)^‡^	0.11	0.21	0.01	0.28
Vitamin K (μg/day)^‡^	0.16	0.35*	0.28	0.43**
Vitamins B1 (mg/day)^‡^	0.48**	0.37*	0.02	0.22
Vitamins B2 (mg/day)^‡^	0.24	0.29	0.33*	0.37*
Niacin (mg/day)^‡^	0.22	0.34*	0.34*	0.38*
Vitamin B6 (mg/day)^‡^	0.33*	0.50**	0.24	0.38*
Vitamin B12 (μg/day)^‡^	0.21	0.32*	0.31	0.25
Folic acid (μg/day)^‡^	0.18	0.46**	0.16	0.43**
Pantothenic acid (mg/day)^‡^	0.26	0.33*	0.29	0.23
Vitamins C (mg/day)^‡^	0.25	0.28	0.24	0.51**
Cholesterol (mg/day)^‡^	0.09	0.29*	0.20	0.09
Dietary fiber (g/day)^‡^	0.51**	0.61**	0.21	0.42**
Saturated fatty acid (g/day)^‡^	0.54**	0.31*	0.02	0.32*
Monounsaturated fatty acid (g/day)^‡^	0.35*	0.19	-0.13	0.02
Polyunsaturated fatty acid (g/day)^‡^	0.13	0.16	-0.09	0.23
n-3 polyunsaturated fatty acid (mg/day)^‡^	0.33*	0.33*	0.09	0.35*
n-6 polyunsaturated fatty acid (mg/day)^‡^	0.18	0.15	-0.09	0.19
Myristic acid (mg/day)^‡^	0.64**	0.18	0.35*	0.63**
Oleic acid (mg/day)^‡^	0.37*	0.19	-0.13	0.01
Linoleic acid (mg/day)^‡^	0.17	0.16	-0.09	0.20
Alpha-linolenic acid (mg/day)^‡^	0.11	0.01	-0.06	0.24
Arachidonic acid (mg/day)^‡^	0.45**	0.22	0.17	0.03
Icosapentaenoic acid (mg/day)^‡^	0.44**	0.39**	0.27	0.34*
Docosahexaenoic acid (mg/day)^‡^	0.38**	0.35*	0.29	0.31

a: Unadjusted Pearson correlation coefficient

b: Nutrient intakes were adjusted for age, sex and energy

‡: The data (Alpha tocopherol, Vitamin K, Vitamin B1, Vitamin B2, Niacin, Vitamin B6, and Vitamin B12) were transformed by natural log to improve normality

CFFQ: Food frequency questionnaire for dietary assessment in children

AFFQ: Food frequency questionnaire for dietary assessment in adults

WDR: Weighed dietary record

SD: Standard deviation

*p < 0.05, **: p < 0.01

### Comparison of correlation coefficients for the different FFQs

We classified the nutrients and fatty acids by their correlation coefficient in adjusted values into 3 categories, 'less than 0.30' (low), '0.30 or more but less than 0.50' (medium) and '0.50 or more' (high) based on the CFFQ and AFFQ. For the CFFQ, the number of nutrients classified as low, medium, and high was 11, 19 and 9 in the YC group, and 16, 18 and 5 in the AD group, respectively. And for the AFFQ, the number of nutrients was 19, 15 and 5 in the YC group and 21, 15 and 3 in the AD group.

### Bland Altman analysis

**Figure 2:**
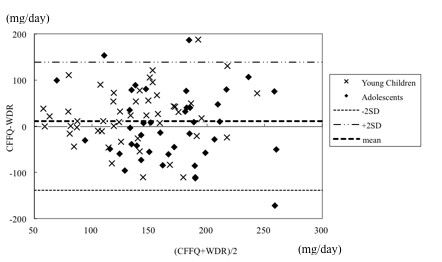
**Agreement of the estimates of arachidonic acid intake between the four WDRs and the CFFQ for all subjects**. CFFQ: Food frequency questionnaire for dietary assessment in children. WDR: Weighed dietary record. SD: Standard deviation.

**Figure 3:**
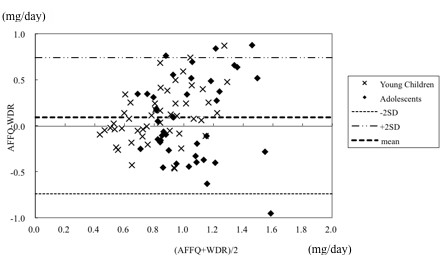
**Agreement of the estimates of vitamin B1 intake between the four WDRs and the AFFQ for all subjects**. AFFQ: Food frequency questionnaire for dietary assessment in adults. WDR: Weighed dietary record. SD: Standard deviation.

The agreements between the WDR and CFFQ or the WDR and AFFQ were assessed using Bland Altman analysis (Figures 2 and 3). The intake of energy and 18 nutrients (carbohydrate, potassium, zinc, copper, vitamin D, alpha tocopherol, vitamin K, vitamin B1, vitamin B2, vitamin B6, folic acid, monounsaturated fatty acid, n-3 polyunsaturated fatty acid, oleic acid, linoleic acid, alpha-linolenic acid, and arachidonic acid) in the CFFQ and the intake of energy and 11 nutrients (protein, carbohydrate, zinc, copper, vitamin D, alpha tocopherol, vitamin K, vitamins B1, folic acid, vitamin C) in the AFFQ showed agreement between the two methods.

## Discussion

Correlation coefficients on the order of 0.5 to 0.7 appear to be typical for the reproducibility of nutrient intake [15]. Cullen et al. (r = 0.21-0.72) [16] and Teiber et al. (r = 0.42-0.74) [17] reported reproducibility of FFQs for children. In our study, the correlation coefficients between the first and second FFQs were higher (r = 0.76 in CFFQ, r = 0.73 in AFFQ) than those in their reports.

We had assumed that the CFFQ would be a more suitable method for assessing children's diets than the AFFQ. The median of the adjusted values between the WDRs and CFFQ were r = 0.39 in the YC group and r = 0.34 in the AD group. The value between the WDRs and AFFQ were r = 0.30 and r = 0.29, respectively. The same moderate correlations were reported by Rockett et al. (r = 0.21-0.58) [2], Marshall et al. (r = 0.20-0.52) [18], and Blum et al. (r = 0.26-0.63) [19]. When we classified the energy and nutrients by their correlation coefficients in adjusted values into 3 categories, for the YC group, the number of nutrients classified as medium and high was 28 in the CFFQ and 20 in the AFFQ. For the AD group, the number was 23 and 18, respectively. Comparing the CFFQ and AFFQ, the CFFQ was more suitable than the AFFQ in both the YC and AD group.

In our study, we found a small difference between the YC and AD groups in terms of the CFFQ or AFFQ.Cullen et al. compared the validity of the FFQ between a group of 10 to 12-year-old children and 13 to 17-year-old adolescents [16]. They reported that the validity was higher in the 13 to 17-year-old group, probably because knowledge about foods or dishes was greater among the older subjects. However, we obtained higher median adjusted values between the WDRs and CFFQ or the WDRs and AFFQ in the YC group than in the AD group. The reason for such differences might have been because the mother completed both the WDRs and FFQs for the subjects in the YC group, while in the AD group, although FFQs were completed by the subjects, the WDRs were completed by either the subjects or their mothers. Because we considered that the subjects in the AD group had more opportunities to eat foods and snacks unknown to their guardians/parents than those in the YC group, we asked the subjects in the AD group to complete the FFQs. However, it might be possible that they had less knowledge about intake frequencies and the portion sizes of the dishes or foods than the mothers in the YC group. To maintain the quality of the data, the registered dieticians asked to the subjects to clarify any unclear answers.

We obtained different correlation coefficients for the same nutrients from the CFFQ and AFFQ, including sodium in the YC group (r = 0.17 in the CFFQ and r = 0.41 in the AFFQ). It is necessary to develop specific FFQs for the group being studied (to accurately reflect socioeconomic, cultural, and seasonal differences). The AFFQ was developed based on the dietary data of adults who lived in several different areas of Japan. Therefore, the AFFQ might have sufficiently covered the dishes and individual foods consumed by subjects in this study. However, the AFFQ was found to have lower correlation coefficients of total fat and fatty acids than the CFFQ, especially in the YC group. It may be necessary to modify a food list in the AFFQ to add foods influencing fat that are regularly eaten by children, and are familiar to children, such as snacks.

Generally, a FFQ originally developed for adults is applied for the assessment of dietary intake in children, however it has been reported that when the FFQ for adults is used, the dietary intake of children is overestimated [5-7]. Fumagalli et al. [5] studied the validity of a FFQ for 5 to 10 year old children, and Wilson [6] studied the validity of a FFQ for 4 to 9 year old children. These authors compared the improved FFQs for adults with 3-day WDRs. They reported that energy and nutrient intakes in the FFQs were overestimated. In our study, we compared the differences in nutrient intake between the WDRs and FFQs. The numbers of over/underestimated nutrients were lower from the CFFQ in the YC group and from the AFFQ in the AD group. The portion size of a FFQ should be suitable for the subjects. Because steamed rice is a particularly important energy source in Japan, it is important to determine the intake of steamed rice as accurately as possible, and three sizes of rice bowls (large, medium, and small) were included in the questionnaire in both the CFFQ and AFFQ (150 g, 120 g, and 80 g in the CFFQ and 200 g, 150 g and 100 g in the AFFQ). The portion size of the CFFQ is appropriate for 3 to 11 year old children, because the CFFQ was developed for this age-group children. Since the AFFQ was developed for adults, the portion size of the AFFQ might be more suitable for the subjects in the AD group who were bigger than those in the YC group.

The values of sodium intake in the CFFQ were underestimated compared to the WDR. In contrast, the values in the AFFQ were overestimated in both the YC and AD groups. The systematic errors were found. For example, when we collected dietary data from children for developing the CFFQ, the subjects' parents/guardians freely decided the day for the WRD [10]. Although we asked subject's parents/guardians to have the children eat habitual diets on the day they selected for the WRD, it was possible that a special day (e.g. a holiday or other celebration where special foods are eaten) may have been selected. Therefore, the food list of the CFFQ might have posed the systematic errors.

In order to assess habitual intake, dietary surveys need to be conducted for several days [20]. However, the WDR is considered a burden some for subjects. In addition, it has been reported that long-term dietary surveys are not always accurate [21]. Therefore, we decided that the mean intake recorded in four WDRs, which were conducted on different days of the week, represented children's habitual dietary intake for one month.

In this study, the two types of FFQs, (i.e. CFFQ and AFFQ) were answered at the same time. We eliminated the any bias which may be caused by administration of the CFFQ and AFFQ by assuring that the order in which each FFQ was answered was randomly determined.

We were not able to assemble a large number of subjects of each sex and age. Therefore, we were not able to calculate the correlation coefficients by sex and age. For the growing children, it is thought that the amount and quality of the dietary intake differs by sex and age. Thus, we have adjusted for sex and age, in addition to energy, for correlation coefficients between the WDR and second FFQ.

We were not able to select subject candidates by random sampling because we had asked the teachers at each school to select participants. The four-day WDRs and 4 FFQs were a heavy burden for the subjects and their parents/guardians. To conduct a dietary survey at the kindergarten, elementary school, and secondary school, the cooperation of the teacher of each school was necessary to ensure that the parents/guardians understood the study. Therefore, the subjects and their mothers who participated in this study might have had more interest in their diets than the general population. In addition, because we specified the days of the dietary survey for subjects and their mothers, it was possible that the subjects may have eaten special meals on those days. Therefore, when we explained the study method to them, we asked them to eat habitual diets on the days of the dietary survey.

The CFFQ does not encompass the seasonal variation in the 75 food items, because when we developed the CFFQ from WDRs collected during only one season, between May and July [10]. To address this potential short-coming, we decided to add seasonal fruits for this study. However, this study was conducted in the only one season. We need the further study to assess the seasonal intakes.

To our knowledge, this is the first study to examine the reproducibility and validity of an FFQ including questions about both individual food items and mixed dishes in 3 to 16 year old Japanese children. The CFFQ might be a useful tool for assessing the habitual dietary intake of 3 to 11 year old children for epidemiologic studies in Japan. In order to more accurately estimate the dietary intake of 12 to 16 year old children, it is necessary to develop a new FFQ or further modify the existing AFFQ or CFFQ.

## Conclusions

This study indicated that the CFFQ might be a useful tool for assessing habitual dietary intake of young children (ages 3-11). Although the CFFQ agreed moderately with habitual intakes, it was found to underestimate dietary intake in the AD group. In addition, using the AFFQ had a low ability to rank habitual intake. Therefore, it is necessary to design a new FFQ or modify an existing FFQ to accurately assess the habitual diet of children aged 12-16.

## Competing interests

The authors declare that they have no competing interests.

## Authors' contributions

TK conceptualized the design of the study and study protocol, as well as performed data collection, statistical analyses and writing of the manuscript. MK, SI, CT and NO contributed to data collection. MF performed statistical analyses. CD contributed to the concept and design of the study and study protocol, as well as performed data collection and assisted in writing and editing the manuscript. All the authors read and approved the final manuscript.

## References

[B1] Mikkilä V Räsänen L Raitakari OT Pietinen P Viikari J Consistent dietary patterns identified from childhood to adulthood: the cardiovascular risk in Young Finns Study Br J Nutr 2005 93 923 931 16022763 10.1079/bjn20051418

[B2] Rockett HR Colditz GA Assessing diets of children and adolescents Am J ClinNutr 1997 65 1116S 1122S 10.1093/ajcn/65.4.1116S 9094907

[B3] Biró G Hulshof KF Ovesen L Amorim Cruz JA EFCOSUM Group Selection of methodology to assess food intake Eur J ClinNutr 2002 56 S25 S32 10.1038/sj.ejcn.1601426 12082515

[B4] Livingstone MB Robson PJ Measurement of dietary intake in children ProcNutrSoc 2000 59 279 293 10.1017/s0029665100000318 10946797

[B5] Fumagalli F Pontes Monteiro J Sartorelli DS Vieira MN de Lourdes Pires Bianchi M Validation of a food frequency questionnaire for assessing dietary nutrients in Brazilian children 5 to 10 years of age Nutrition 2008 24 427 432 10.1016/j.nut.2008.01.008 18343639

[B6] Wilson AM Lewis RD Disagreement of energy and macronutrient intakes estimated from a food frequency questionnaire and 3-day diet record in girls 4 to 9 years of age J Am Diet Assoc 2004 104 373 378 10.1016/j.jada.2003.12.021 14993859

[B7] Serdula MK Alexander MP Scanlon KS Bowman BA What are preschool children eating? A review of dietary assessment Annu Rev Nutr 2001 21 475 498 10.1146/annurev.nutr.21.1.475 11375446

[B8] Bertoli S Petroni ML Pagliato E Mora S Weber G Chiumello G Testolin G Validation of food frequency questionnaire for assessing dietary macronutrients and calcium intake in Italian children and adolescents J PediatrGastroenterolNutr 2005 40 555 560 10.1097/01.mpg.0000153004.53610.0e 15861015

[B9] Wakai K A review of food frequency questionnaires developed and validated in Japan J Epidemiol 2009 19 1 11 10.2188/jea.JE20081007 19164867 PMC3924089

[B10] Kobayashi T Tanaka S Toji C Kamimura M Okamoto N Imai S Fukui M Date C Development of a food frequency questionnaire to estimate habitual dietary intake in Japanese children Nutr J 2010 9 17 10.1186/1475-2891-9-17 20380735 PMC2873262

[B11] Date C Yamaguchi M Tanaka H Development of a food frequency questionnaire in Japan J Epidemiol 1997 6 131S 136S 10.2188/jea.6.3sup_131 8800285

[B12] Ministry of Education, Culture, Sports, Science and Technology Standard tables of food composition in Japan fifth revised and enlarged edition 2005 Tokyo: National Printing Bureau (in Japanese)

[B13] Willett W Stanpfer M Willett W Implications of total energy intake for epidemiologic analyses Nutritional epidemiology 1998 2 Oxford: Oxford University Press 273 301 full_text

[B14] Bland JM Altman DG Statistical methods for assessing agreement between two methods of clinical measurement Lancet 1986 1 307 310 10.1016/S0140-6736(86)90837-8 2868172

[B15] Willett W Lenart E Willett W Reproducibility and validity of Food-frequency questionnaires Nutritional epidemiology 1998 2 Oxford: Oxford University Press 101 147 full_text

[B16] Cullen KW Watson K Zakeri I Relative reliability and validity of the Block kids questionnaire among youth aged 10 to 17 years J Am Diet Assoc 2008 108 862 866 10.1016/j.jada.2008.02.015 18442512

[B17] Treiber FA Leonard SB Frank G Musante L Davis H Strong WB Levy M Dietary assessment instruments for preschool children: reliability of parental responses to the 24-hour recall and a food frequency questionnaire J Am Diet Assoc 1990 90 814 820 2345254

[B18] Marshall TA Eichenberger Gilmore JM Broffitt B Stumbo PJ Levy SM Relative validity of the Iowa Fluoride Study targeted nutrient semi-quantitative questionnaire and the Block kids' food questionnaire for estimating beverage, calcium, and vitamin D intakes by children J Am Diet Assoc 2008 108 465 472 10.1016/j.jada.2007.12.002 18313429

[B19] Blum RE Wei EK Rockett HR Langeliers JD Leppert J Gardner JD Colditz GA Validation of a food frequency questionnaire in Native American and Caucasian children 1 to 5 years of age Matern Child Health J 1999 3 167 172 10.1023/A:1022350023163 10746756

[B20] Egami I Wakai K Kaitoh K Kawamura T Tamakoshi A Lin Y Nakayama T Sugimoto K Ohno Y Intra- and inter-individual variations in diets of the middle-aged and the elderly Nippon KoshuEiseiZasshi 1999 46 828 837 (in Japanese) 10540854

[B21] Willett WC Sampson L Stampfer MJ Rosner B Bain C Witschi J Hennekens CH Speizer FE Reproducibility and validity of a semiquantitative food frequency questionnaire Am J Epidemiol 1985 122 51 65 4014201 10.1093/oxfordjournals.aje.a114086

